# Mucin dysregulation in salivary gland tumorigenesis: Diagnostic, prognostic, and therapeutic insights: A systematic review & meta-analysis

**DOI:** 10.1016/j.jobcr.2026.101533

**Published:** 2026-09-09

**Authors:** Shreya Chatterjee, Gopikrishnan Vijayakumar, Rahul Anand, Charu Priya, Shanvi Kumari

**Affiliations:** a Rajendra Institute of Medical Sciences, Ranchi, Jharkhand, 834009, India; b Dept. of Oral Maxillofacial Pathology &Oral Microbiology, Tirumala Institute of Dental Sciences and Research Centre, Nizamabad, Telangana, 503230, India; c Dept. of Oral Maxillofacial Pathology & Oral Microbiology, Dr. D.Y. Patil Vidyapeeth, Pimpri, Pune, Maharashtra, 411018, India; d Dept. of Cardiology, All India Institute of Medical Sciences, New Delhi, 110029, India; e Oral & Maxillofacial Pathology & Oral Microbiology, Subharat Dental Clinic, Patna, Bihar, 801503, India

**Keywords:** Mucin, Salivary gland tumors, MUC1, MEC, PA, Diagnostic utility

## Abstract

**Background:**

Salivary gland tumors (SGTs) comprise a heterogeneous group of neoplasms with overlapping histopathological features, making diagnosis and classification challenging Mucins(MUCs) are high molecular weight glycoproteins involved in epithelial differentiation and tumorigenesis which have emerged as potential biomarkers in salivary gland pathology.

**Methodology:**

A thorough review and meta-analysis were conducted across PubMed, Scopus, Web of Science, and Embase until February 2025 to elucidate the diagnostic, prognostic, and therapeutic significance of mucin differential expression in benign and malignant SGTs.

**Result:**

Twenty-nine articles encompassing 1657 salivary gland cases were included. Data on mucins, their localization, and expression pattern using immunohistochemistry, RT-PCR, or ELISA were evaluated. MUC1 and MUC4 were predominantly expressed in PA and MEC, with MUC1 associated with high-grade MECs and recurrence in PA. MUC4 showed higher expression in low-grade MECs and was diagnostically specific for secretory carcinoma. MUC1 showed high expression in both MEC and PA, but the pooled odds ratios [MEC: OR = 2.23; PA: OR = 0.35] were statistically non-significant (p > 0.05), with moderate heterogeneity (I^2^ = 53% for MEC) and poor subgroup differences. MUC4 had a sensitivity of 81% but exhibited near-zero specificity (0%), and its odds ratio (0.85 [95% CI: 0.17–4.30]) lacked statistical significance.

**Conclusion:**

Mucins demonstrate differential expression across salivary gland tumors and may complement histopathological evaluation. However, the current quantitative evidence remains insufficient to support their use as standalone diagnostic biomarkers due to limited specificity and considerable uncertainty in pooled estimates.

## Highlights

• Salivary gland tumors (SGTs) are morphologically diverse, and traditional histological classification systems are often insufficient for predicting tumour behaviour, recurrence, or therapeutic response, necessitating novel biomarkers like mucins.
• Mucins, including both secreted and membrane-bound forms, demonstrate altered expression, localization, and glycosylation profiles in SGTs, with differential expression patterns holding diagnostic, prognostic, and therapeutic implications.
• While MUC1 is frequently expressed in mucoepidermoid carcinoma (MEC) and pleomorphic adenoma (PA), its high sensitivity but low specificity limits its diagnostic utility in distinguishing between MEC, PA, and normal salivary glands (NSG).
• MUC4 expression is strongly associated with secretory carcinoma (SeC), exhibiting high sensitivity (100%) and specificity (90.7%), making it a valuable diagnostic marker for this specific subtype.
• This systematic review and meta-analysis highlight a need for standardized mucin detection methods and scoring criteria across research, as methodological variability introduces heterogeneity and complicates the interpretation of diagnostic and prognostic values.

## 1 Introduction

Salivary gland tumors (SGTs) encompass a morphologically diverse group of benign and malignant neoplasms arising from the major or minor salivary glands, exhibiting variable biological behavior, cellular composition, and clinical outcomes.^1^ The majority of SGTs are benign, with the major salivary glands accounting for around 70% of cases and the minor salivary glands for about 25%. An estimated 2–6% of all head and neck cancers are malignant SGTs.^2^^,^^3^ Despite their relatively low incidence, SGTs present significant diagnostic challenges due to their varied histological presentation and overlapping morphological features, especially among low-grade malignancies and benign mimics.^4^ Novel biomarkers are needed to complement morphological evaluation since traditional classification systems based on histological architecture are frequently inadequate for precisely predicting tumourbehaviour, recurrence, or treatment response.^5^

Diagnostic difficulties frequently arise in distinguishing low-grade mucoepidermoid carcinoma from benign cystic lesions, secretory carcinoma from acinic cell carcinoma, and polymorphous adenocarcinoma from adenoid cystic carcinoma, particularly in small biopsy specimens.^4^^,^^5^ In such situations, morphology alone may be insufficient for definitive diagnosis, highlighting the need for reliable ancillary biomarkers that can complement routine histopathological assessment and improve diagnostic accuracy.

Among the emerging candidates, mucins (MUCs) are a family of high molecular weight, glycosylated proteins which have garnered attention for their multifaceted roles in epithelial homeostasis, tumorigenesis, and immune modulation.^6^ Over the past decades, mucin evaluation has evolved from conventional histochemical stains to immunohistochemical and molecular techniques, enabling more accurate characterization of mucin expression patterns in salivary gland tumors. Mucins are broadly divided into secreted (gel-forming) and membrane-bound forms, each contributing differently to the structural and functional integrity of glandular epithelia and exhibits unique alterations in neoplastic environments.^7^ Membrane-bound mucins like MUC1 and MUC4 are involved in intracellular signalling, cell polarity, and adhesion, whereas secreted mucins like MUC2, MUC5AC, MUC5B, and MUC6 are often involved in mucosal lubrication and defence.^8^

In normal salivary glands, mucin expression is highly compartmentalized: MUC1 and MUC4 are largely localized to ductal epithelial cells, while MUC5B and MUC7 are predominantly expressed in mucous and serous acinar cells, respectively.^9^ However, in salivary gland tumors, this compartmentalization is disrupted, with significant shifts in expression levels, localization patterns, and glycosylation profiles depending on the histological subtype, grade, and malignant potential.^1^

Differential mucin expression has diagnostic, prognostic, and potentially therapeutic implications in SGTs.^9^ Its overexpression has also been documented in pleomorphic adenomas and carcinomas ex-pleomorphic adenoma, where it may reflect the tumor's invasive potential and recurrence risk.^1^ Conversely, MUC4 expression is generally linked to lower-grade MECs and more favorable clinical outcomes, emphasizing its potential as a prognostic indicator.^7^

Immunohistochemical analysis has also defined these patterns further with findings that MUC1 and MUC5AC are more commonly expressed in Mucoepidermoid Carcinoma than in adjacent non-neoplastic salivary tissue indicating neoplasm-specific upregulation.^6^ From a histogenetic perspective, distinct paths of tumour development and lineage commitment may be reflected in the diverse mucin expression across the various tumour cell types, including epidermoid, mucous, intermediate, and glandular.^4^ The aforementioned strategy is in accordance with the hypothesis that salivary gland tumors originate from reserve or progenitor cells in the excretory ducts, which may result in a spectrum of phenotypic manifestations, including aberrant mucin synthesis, depending on genetic changes and microenvironmental stimuli.^5^

Beyond their biological significance, differential mucin expression has attracted increasing attention as a potential diagnostic and prognostic adjunct in salivary gland pathology. However, despite growing evidence regarding the role of mucins in tumour classification, progression, and clinical outcomes, the available literature remains fragmented and occasionally inconsistent, particularly with respect to the diagnostic and prognostic utility of MUC1 and MUC4 across different salivary gland tumour subtypes.

Although several narrative reviews have explored mucin biology in salivary gland neoplasms, no systematic review and meta-analysis has comprehensively synthesized the available evidence regarding mucin dysregulation across benign and malignant salivary gland tumors. Moreover, recent studies have reported variable findings concerning the diagnostic and prognostic significance of MUC1 and MUC4, particularly in mucoepidermoid carcinoma and secretory carcinoma. Therefore, an updated evidence synthesis is necessary to consolidate current knowledge, evaluate diagnostic performance, and identify future research priorities.

Thus, further evidence synthesis is required to clarify that standard diagnostic procedures may enhance histopathological classification, enabling early diagnosis, and, potentially guide targeted therapy in selected cases. This systematic review seeks to consolidate current evidence on mucin expression in salivary gland tumors, highlighting their diagnostic, prognostic, and therapeutic relevance across the spectrum of salivary gland tumors.

## 2 Material and methods

### 2.1 Research question & study protocol

Based on ‘PICO’ (Population, Intervention, Comparison, Outcome), the review question was formulated as follows: ‘Do mucins exhibit differential expression in various salivary gland tumors? ‘The study protocol was designed to adhere to the Preferred Reporting Items for Systematic Reviews and Meta-Analyses (PRISMA) guidelines and was registered in the Prospective International Registration of Systematic Reviews database (Reference number CRD42025636208).

### 2.2 Search strategy

We performed an electronic search on PubMed (Medline), Scopus, Web of Science and Embase databases, until 1st February 2025 without period restriction. The search strategy was based on Medical Subject Headings (MeSH) and a combination of free words in the title/abstract as follows: (salivary gland tumor[Title/Abstract] OR carcinoma of the salivary gland[Title/Abstract] OR salivary gland cancer[Title/Abstract]) AND (mucins or MUC1 or MUC2 or MUC3 or MUC4, MUC5ACor MUC5Bor MUC6 or MUC7 or MUC8 or MUC12, MUC13, MUC16, MUC17, MUC18, MUC19). The search syntaxes were modified to match each search engine's usage. Strict inclusion and exclusion criteria were applied during a manual search of papers.

#### 2.2.1 Inclusion criteria

1. Study Type:
  ○ Original research articles (cohort studies, case-control studies, cross-sectional studies).
  ○ Studies with immunohistochemical, molecular, or histopathological analysis of mucins.
2. Population:
  ○ Patients diagnosed with benign or malignant salivary gland tumors.
  ○ Studies including normal salivary gland tissue as a control group were also included.
3. Mucin Types:
  ○ Studies evaluating any mucin type (MUC1, MUC2, MUC4, MUC5AC, MUC5B, MUC7, etc.) or isoforms of the antibody against any of the mentioned mucins.
4. Language and Publication:
  ○ Peer-reviewed articles published in English.

#### 2.2.2 Exclusion criteria

1. Study Type:
  ○ Case reports, review articles, meta-analyses, conference abstracts, editorials.
  ○ Animal or in vitro studies without human tissue validation.
2. Population:
  ○ Studies without histologically confirmed salivary gland tumour diagnosis.
  ○ Studies with a mixed tumour population where salivary gland tumors are not analyzed separately.
3. Data Availability:
  ○ Studies lacking sufficient diagnostic data (e.g., missing sensitivity/specificity, AUC, or other diagnostic measures).
  ○ Incomplete or irretrievable data.
4. Language:
  ○ Non-English publications (unless a reliable translation is available).

### 2.3 Data extraction

With the objective to weed out irrelevant data, two reviewers (Authors 1 and 2) independently assessed the titles and abstracts of articles identified via the search approach. A third reviewer resolved any discrepancies between the two reviewers. Decisions were recorded into a Microsoft Excel spreadsheet. The following data were extracted from the included studies: first author, year of publication, population origin, number of patients, clinical and pathological features, tumour subtype, technology employed, nature of mucin studies, and cut-off scores.

### 2.4 Quality assessment

The risk of bias in the included articles was evaluated using the Quality Assessment in Diagnostic Accuracy (QUADAS 2) tool, and the results were generated using Review Manager (RevMan v5.3, The Nordic Cochrane Centre, The Cochrane Collaboration, Copenhagen, Denmark). Patient selection, flow and timing, index test, and reference standard were the checklist elements used to assess risk bias and applicability concerns. Two authors (Author 1 and Author 2) independently assessed the risk of bias on each tool domain; disagreements were resolved through discussion.

### 2.5 Summary measures, data synthesis and analysis

The evaluation of mucin expression in SGTs was the primary outcome of the review. For quantitative synthesis, mucin expression data were extracted as positive or negative events according to the definitions and cut-off criteria reported in the original studies. Where studies employed ordinal immunohistochemical scoring systems, expression categories were dichotomized based on the authors' predefined thresholds to facilitate pooled analysis.

The meta-analysis was performed using Review Manager (RevMan v5.3, The Nordic Cochrane Centre, The Cochrane Collaboration, Copenhagen, Denmark) software. For each eligible comparison, two-by-two contingency tables were reconstructed from the reported data wherever feasible. Odds ratios (ORs) with corresponding 95% confidence intervals (95% CI) were calculated as the summary effect measure.

Given the anticipated methodological and clinical heterogeneity across studies including differences in tumour subtype, immunohistochemical antibody clones, staining protocols, positivity thresholds, and scoring systems a random-effects model was used to generate pooled estimates. Statistical heterogeneity was assessed using Cochran's Q test together with the I^2^ statistic, where values of approximately 25%, 50%, and 75% represented low, moderate, and high heterogeneity, respectively. Forest plots were generated for all pooled analyses. Statistical significance was defined as p < 0.05. Where sufficient data were available, diagnostic accuracy measures were additionally derived to complement the pooled effect estimates. Sensitivity was defined as the proportion of tumour cases correctly identified as mucin-positive, whereas specificity represented the proportion of normal salivary gland tissues correctly identified as mucin-negative.

Owing to the limited number of studies available for each comparison, publication bias assessment using funnel plots was not considered reliable and therefore was not performed.

## 3 Result

### 3.1 Search results

**Fig. 1:**
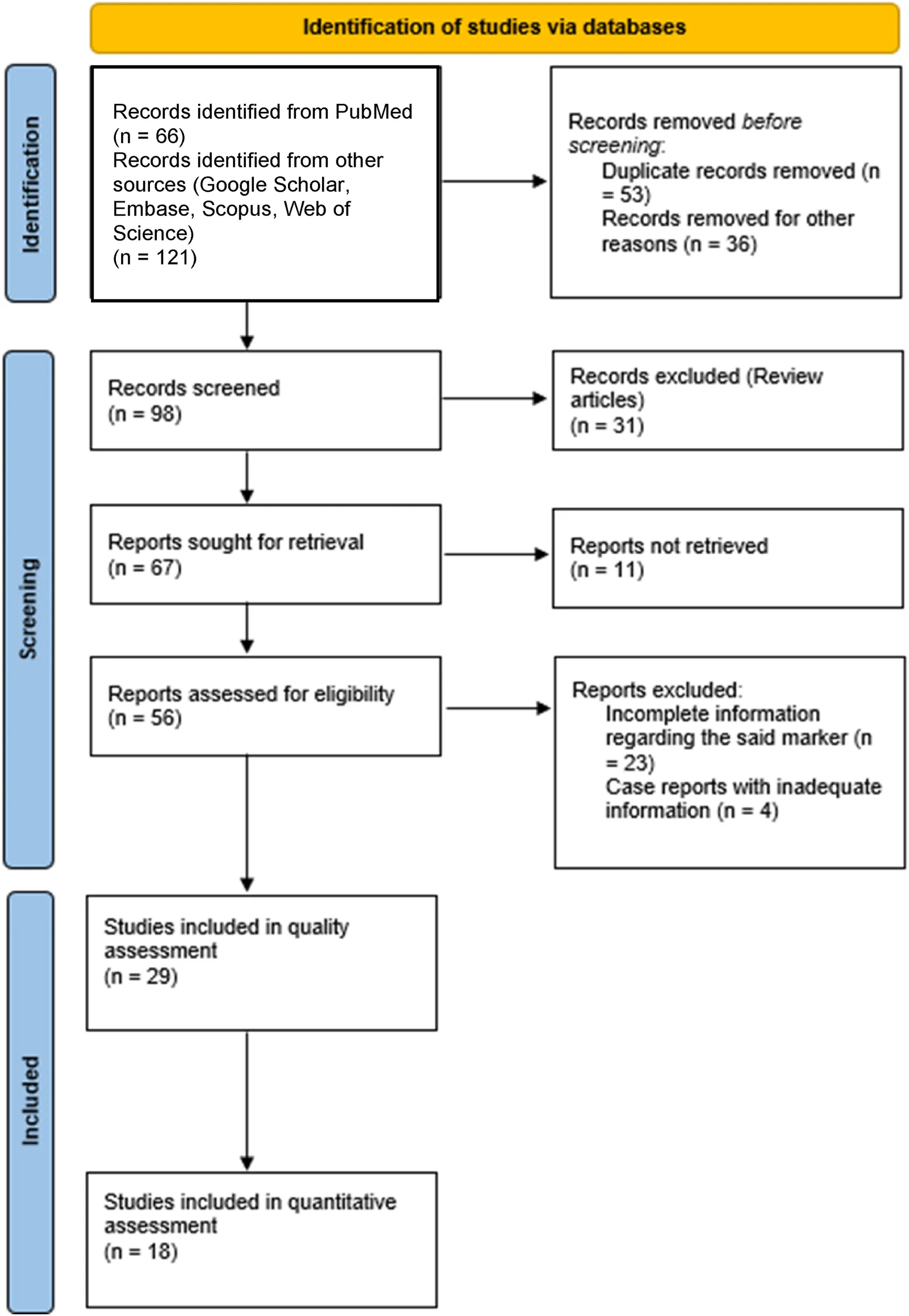
Flow chart of study selection adapted from PRISMA.

The keyword search strategy in various scientific databases identified 187 articles published till 1st February 2025.89 were eliminated from the original pool considering they were either redundant or irrelevant. 31 review articles were eliminated from the 98 assessed records. 11 of the 67 full-text reports that were stipulated were not accessible. 27 of the 56 cases that were evaluated for eligibility were excluded due to insufficient case reports or inadequate data. Based on comprehensive and pertinent data on mucin markers, 18 of the 29 studies that were ultimately included in the qualitative synthesis qualified for a quantitative meta-analysis (Fig. 1).

### 3.2 Study characteristics

The selected 29 studies were conducted in 15 different countries between 1993 and 2023, with five having been conducted in Germany,^5^^,^^10^, ^11^, ^12^, ^13^ two from Portugal,^4^^,^^14^ two from China,^15^^,^^16^ Brazil,^8^^,^^17^ and Mexico,^18^^,^^19^ four from the USA^20^, ^21^, ^22^, ^23^& Japan,^1^^,^^7^^,^^24^^,^^25^ and one each from the Netherlands,^26^ France,^6^ South Korea,^27^ Spain,^9^ India,^28^ South Africa,^29^ and Austria.^30^

We obtained a total of 1657 cases of salivary gland pathologies encompassing 232 benign SGTs, 1283 malignant SGTs, 14 non-neoplastic lesion and salivary cysts and 128 normal salivary glands. 144 pleomorphic adenomas (PA), 40 recurrent pleomorphic adenomas (RPA), 22 Warthin tumors (WT), 11 basal cell adenomas (BCA), 12 papillary cystadenomas (PCA), and three recurrent pleomorphic adenomas with malignant transformation (TRPA) were among the benign tumors.

Within malignant lesions, Mucoepidermoid carcinoma (MEC) was explored the most with 833 cases followed by 121 cases of Salivary duct carcinoma (SDC), 85 acinic cell carcinoma (ACC), 68 Adenoid cystic carcinoma (AdCC), 61 Secretory carcinoma (SeC), 30 Intraductal carcinoma (IDC), 23 Basal cell adenocarcinoma (BCAC), 21 Polymorphous adenocarcinoma (PAC), 14 Mammary analogue salivary carcinoma (MASC), 11 Carcinoma-ex pleomorphic adenoma (Ca-ex-PA), nine Adenocarcinoma not otherwise specified (ADNOS) and seven Epithelial-myoepithelial carcinoma (EMC).

The various mucins studied were MUC1, MUC2, MUC3, MUC4, MUC5AC, MUC5B, MUC6, MUC7, MUC8, MUC12, MUC13, MUC16, MUC17, MUC18 and MUC19. Furthermore, certain isoforms of antibodies against MUC1 have been investigated in the form of human milk fat globules (HMFG1 & 2). Lastly, simple mucin carbohydrate antigens T, Tn, sialosyl-T, and sialosyl-Tn (sTn) were incorporated as antibodies against some of their precursor molecules (Table S1).

### 3.3 Analysis of Mucin expression

In the present review, 27 studies employed immunohistochemical analysis for observing the expression of mucins while one used RT-PCR for studying MUC12, MUC13, MUC17, MUC18, MUC19 and one observed MUC8 expression by ELISA and immunoblotting^11^^,^^21^ Detailed case-wise frequencies and localization patterns of individual mucins are summarized in Table S1.

#### 3.3.1 Mucins in pleomorphic adenoma and other benign SGTs

Pleomorphic adenoma (PA), the most frequently studied benign tumor (144 cases), demonstrated predominant MUC1 and MUC4 expression. MUC1 was observed in 79 cases, showing both membranous and cytoplasmic localization, particularly on the apical membrane of excretory ductal cells. HMFG1/2 positivity was noted in 21 and 23 cases, respectively, indicating expression of specific MUC1 epitopes. MUC2 was detected in 21 cases, typically within luminal cells and excretory ducts.

Recurrent pleomorphic adenoma (RPA) exhibited MUC1 positivity in 27 of 40 cases, with localization similar to PA, suggesting MUC1 as a potential marker of recurrence. Warthin tumors (WT, n = 22) did not express any of the evaluated mucins, consistent with their oncocytic composition and lack of muco-secretory differentiation. Basal cell adenomas (BCA) showed HMFG positivity in 4 of 11 cases, particularly in luminal structures. Papillary cystadenomas (PCA, n = 12) displayed co-expression of MUC1 and MUC4 in 9 cases each, with focal luminal membrane and cytoplasmic staining.

#### 3.3.2 Mucins in mucoepidermoid carcinoma

MEC was the most extensively investigated malignant lesion (833 cases). MUC1 was highly expressed (609 cases), with strong membranous and cytoplasmic positivity across all three cell types—mucous, intermediate, and epidermoid. Several studies reported diffuse cytoplasmic localization in high-grade tumors and apical membranous staining in low/intermediate grades.

234 instances tested positive for MUC4, which is predominantly membranous and cytoplasmic and is particularly expressed in low-grade malignancies. MUC6 (39 cases) has been demonstrated in epidermoid and clear cells, whereas secretory mucins such as MUC5AC(167 cases), MUC5B(56 cases), and MUC2 (23 cases) were mainly localized to mucous and luminal cell populations. Other rare mucins included MUC19 (15 instances, cytoplasmic) and MUC13 (3 cases, cytoplasmic).

Additionally, MEC displayed altered glycosylation patterns: abnormal O-glycan processing was indicated by the expression of T, Tn, sT, and sTn antigens in over 50 cases each, predominantly in intermediate and mucous cells.

#### 3.3.3 Mucins in salivary duct carcinoma

Among 121 SDC cases, focal expression of MUC2, MUC5AC, MUC5B, MUC6, and MUC7 was noted (4 cases each), often localized to mucin lakes and tumor cell cytoplasm. Occasionally, extracellular mucin pools were also recognized, especially with MUC5B and MUC6. These results indicate the generation of mucin in small, dispersed foci.

#### 3.3.4 Mucins in adenoid cystic carcinoma

AdCC (n = 68) expressed MUC1 in 26 cases, primarily along the luminal borders of tubular structures, with some cytoplasmic extension. MUC2 expression was restricted to luminal cells in only 2 cases.

#### 3.3.5 Mucins in acinic cell carcinoma

ACC (n = 85) demonstrated expression of MUC1 (34 cases), MUC2 (12), MUC3 (8), and MUC4 (44). MUC1 was localized along ductal lumina and within secretory acinar structures, while MUC3 showed focal cytoplasmic staining, useful in distinguishing ACC from other neoplasms. MUC4 expression was cytoplasmic and membranous.

#### 3.3.6 Mucins in other malignant SGTs

Secretory carcinoma (SeC) (n = 61) showed strong MUC4 expression in 49 cases, primarily luminal and cytoplasmic, indicating its diagnostic utility. MASC (n = 14) exhibited focal, typically luminal, MUC1 and MUC4 positive (4 instances each). In eight instances, BCAC (n = 23) revealed HMFG expression. All investigated antigens revealed minimal to no mucin expression in lesions such ADNOS, EMC, PAC, Ca-ex-PA, and IDC, implying that mucins in these subtypes have no diagnostic relevance. Specifically, IDC (n = 30) was perpetually negative for all mucin.

#### 3.3.7 Mucins in other lesions

Non-neoplastic lesions (n = 11) revealed positivity for MUC1 and MUC2 in 10 cases each, localized to ductal epithelium and luminal cells. Salivary duct cysts (n = 3) were uniformly positive for MUC4, with membranous and luminal expression in epithelial lining cells.

#### 3.3.8 Mucins in normal salivary gland

Among 147 cases of normal salivary tissue, the most frequent mucins were MUC1 (27 cases), MUC5B and MUC7 (22 each), and MUC4 (20). MUC1 was localized to the apical membranes of excretory ducts, MUC5B to mucous acini, and MUC7 to serous acini. Occasionally, MUC5AC, MUC8, and MUC19 were also noted. Ductal and acinar epithelial cells stained strongly with HMFG1 and HMFG2 (18 instances each). 12 cases each of the carbohydrate antigens T, Tn, sT, and sTn were identified, predominantly in ductal structures.

### 3.4 Quality assessment (risk of bias and applicability concern)

The QUADAS-2 evaluation of 29 studies revealed variability in methodological quality. Within the patient selection domain, 7/29 studies had an unclear risk ^12^^,^^13^^,^^15^^,^^17^^,^^20^^,^^21^^,^^28^ and 1/29 showed high risk.^16^ The index test domain presented high risk in 1/29.^22^ The reference standard domain had high risk in 1/29^20^ and unclear in 1/29.^22^ Flow and timing were marked as unclear in 2/29 studies.^6^^,^^20^

**Fig. 2:**
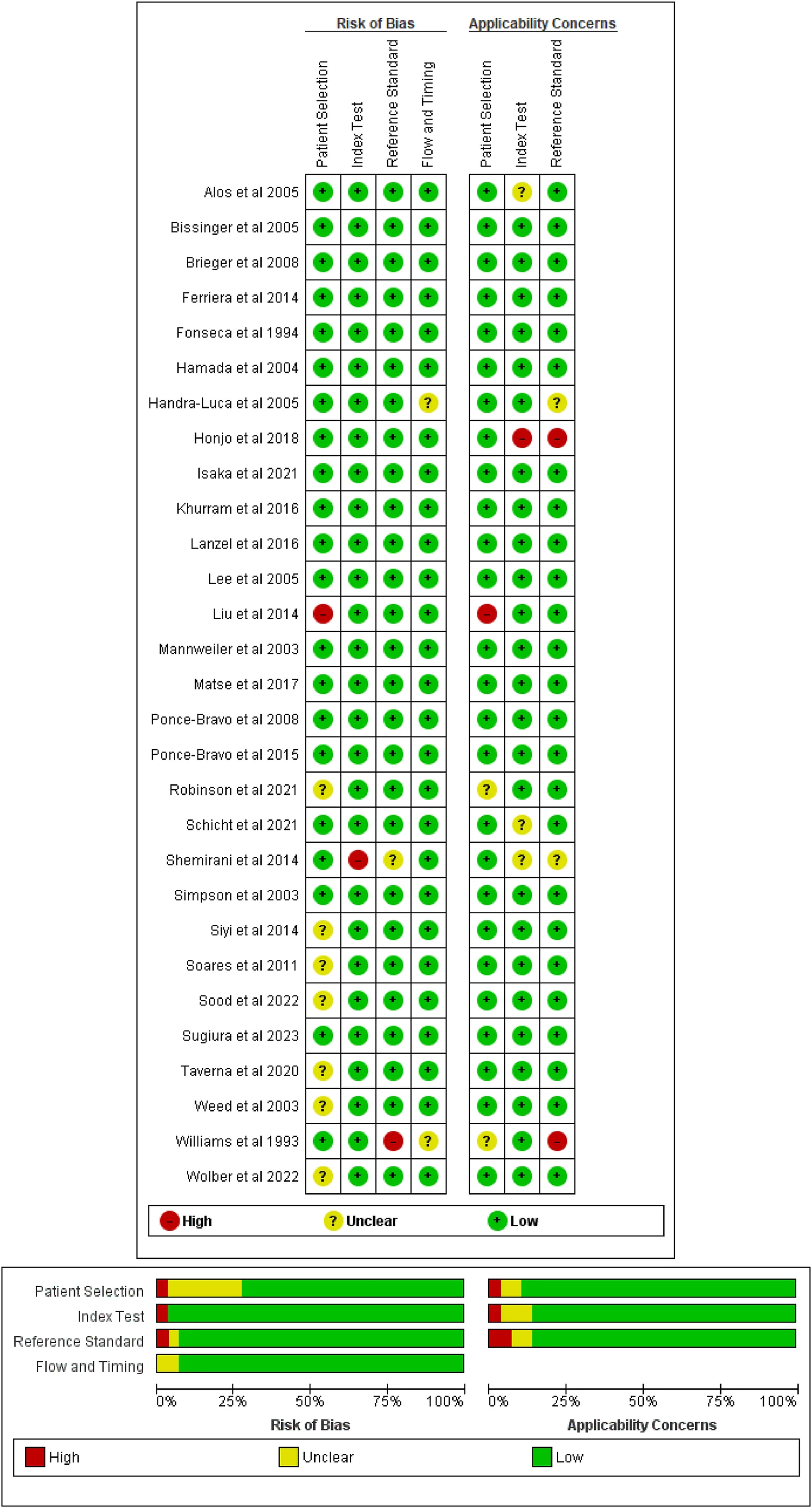
Evaluation of risk of bias of primary-level studies using the quality assessment in diagnostic accuracy (QUADAS 2) tool.

Addressing applicability issues, 1/29 studies had high risk^16^ and 2/29 had ambiguous risk in patient selection.^20^^,^^29^ 3/29 trials^9^^,^^11^^,^^22^ had uncertain risk under the index test domain, while 1/29 had high risk.^7^ Furthermore, in the reference standard domain, 2 out of every 29 studies were reported to have high^7^^,^^20^ and unreliable risk^6^^,^^22^ (Fig. 2).

### 3.5 Meta-analysis of the risk of association

#### 3.5.1 MUC1 expression in mucoepidermoid carcinoma

**Fig. 3:**
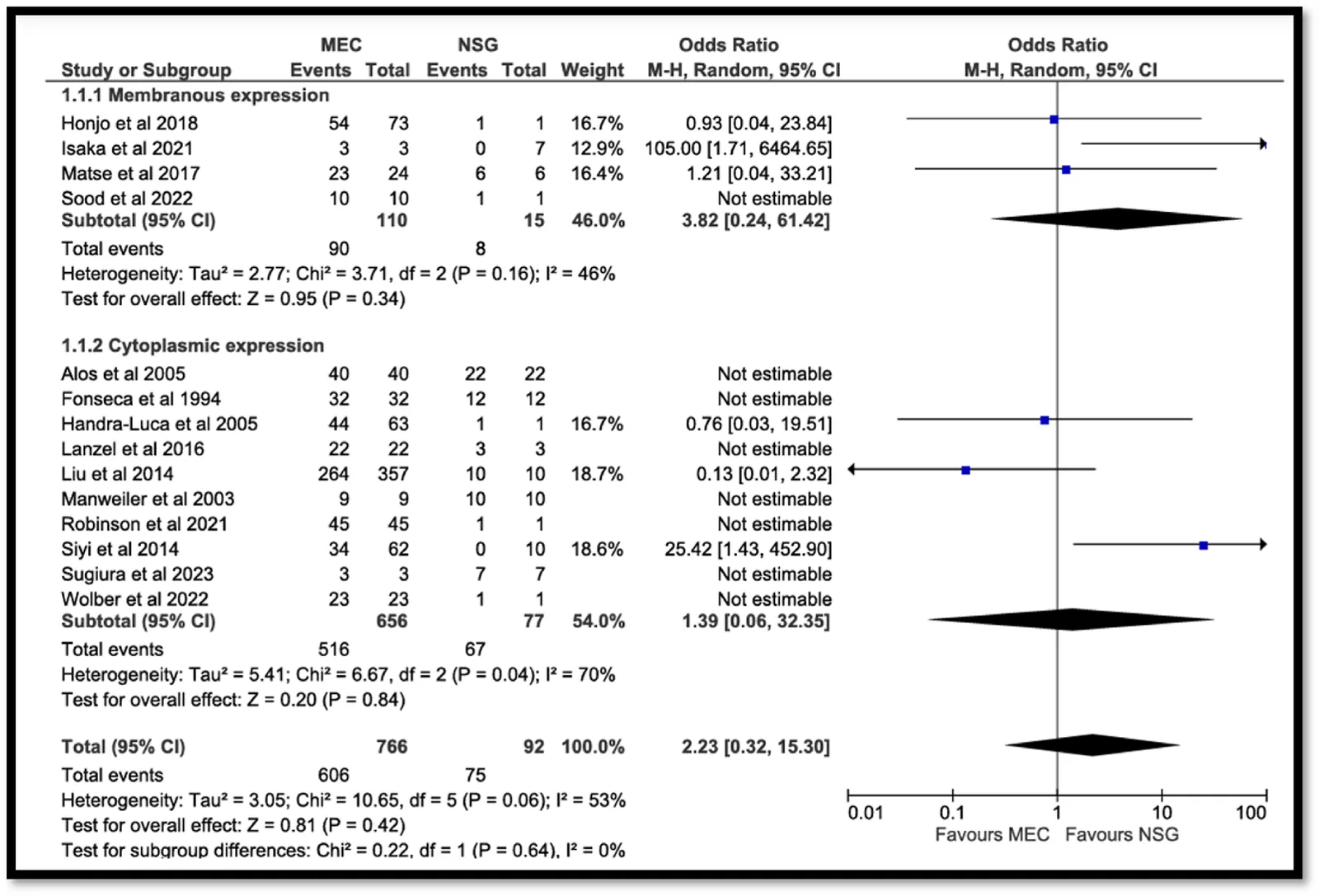
Forest plot showing expression of MUC1 in MEC along with the localization of their expression.

With respect to MUC1 expression localization in MEC, a total of four studies were included in a subgroup meta-analysis. The random-effects model confirmed that higher odds of membranous MUC1 expression in MEC was present (pooled odds ratio 3.82, 95% confidence interval – 0.24 - 61.42, p < 0.05). There was moderate heterogeneity among the studies (I^2^ = 46%, p = 0.9) (Fig. 3). Pertaining to the cytoplasmic expression a total of ten studies indicated a trend toward higher cytoplasmic MUC1 expression in MEC (combined odds ratio 1.39, 95% confidence interval – 0.06–32.35, p < 0.05). There was moderate heterogeneity among the studies (I^2^ = 70%, p = 0.8) (Fig. 3). Therefore, the combined analysis depicts an overall odds ratio of 2.23 (95% confidence interval – 0.32 – 15.30, p = 0.06) and moderate heterogeneity (I^2^ = 53%, p = 0.42) (Fig. 3).

While testing the subgroup difference, there was no significant difference between membranous vs. cytoplasmic expression patterns with null heterogeneity (I^2^ = 0, p = 0.6). MUC1demonstratedhigh sensitivity (79.2%) in detecting MEC (i.e., it's often expressed in MEC), but very low specificity (18.5%) reducing its diagnostic discrimination.

#### 3.5.2 MUC1 expression in pleomorphic adenoma

**Fig. 4:**
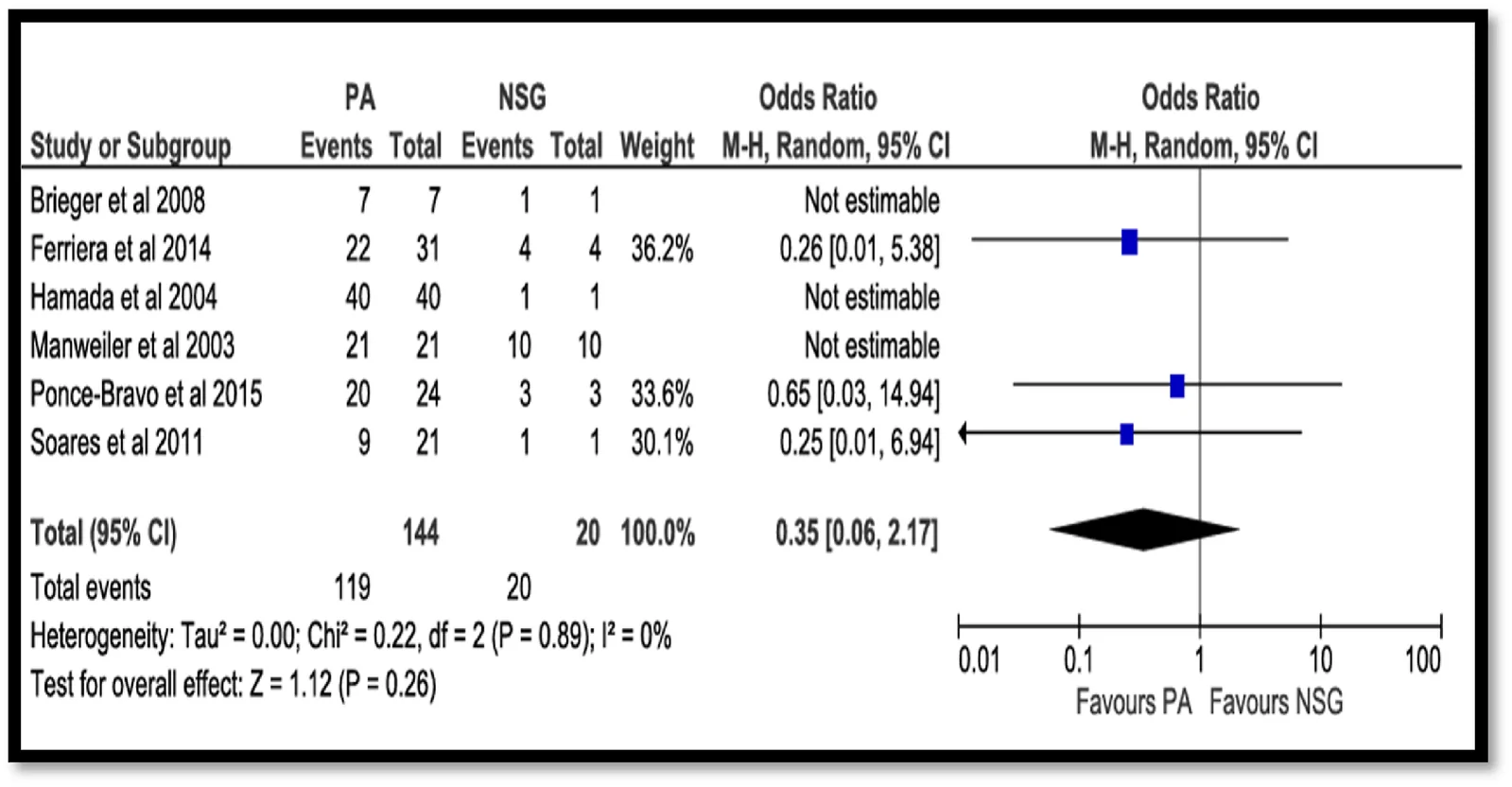
Forest plot showing expression of MUC1 in PA.

The random-effects model demonstrated null heterogeneity (I^2^ = 0%, p = 0.26) and an odds ratio of 0.35 [0.06, 2.17] (p > 0.05) in six studies that were part of the meta-analysis that showed reduced MUC1 expression in PA compared to NSG (Fig. 4). MUC1 is not specific (0%), despite its excellent sensitivity (82.6%) for PA detection.

#### 3.5.3 MUC4 expression in mucoepidermoid carcinoma

**Fig. 5:**
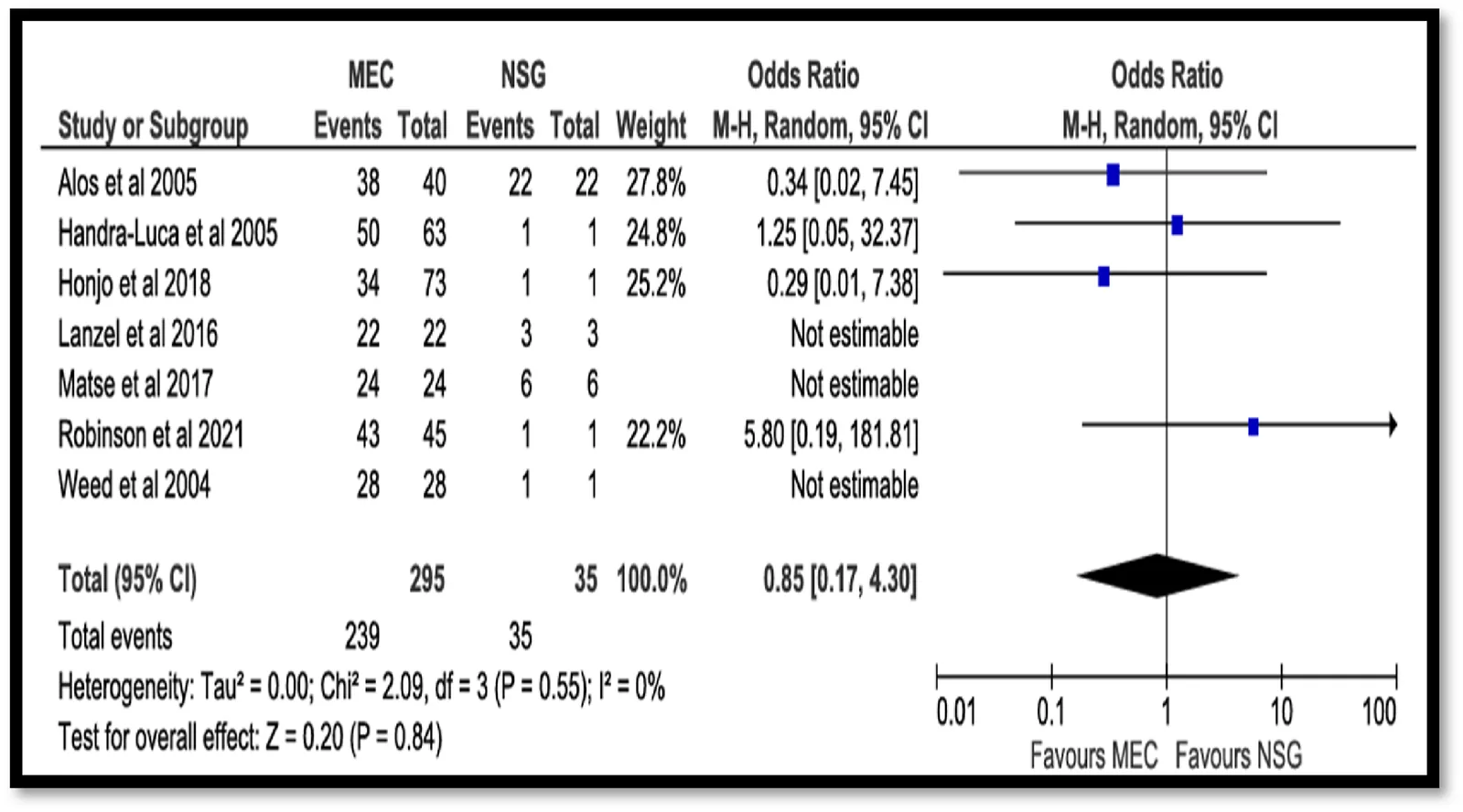
Forest plot showing expression of MUC4 in MEC.

The meta-analysis was conducted with seven studies exhibiting lower MUC4 expression in MEC than in NSG with the random-effects model showing odds ratio of 0.85 [0.17, 4.30] (p > 0.05) and null heterogeneity (I^2^ = 0%, p = 0.84) (Fig. 5). Additionally, MUC4 shows minimal specificity (0%) and high sensitivity (81%) in detecting MEC.

#### 3.5.4 MUC5AC expression in mucoepidermoid carcinoma

**Fig. 6:**
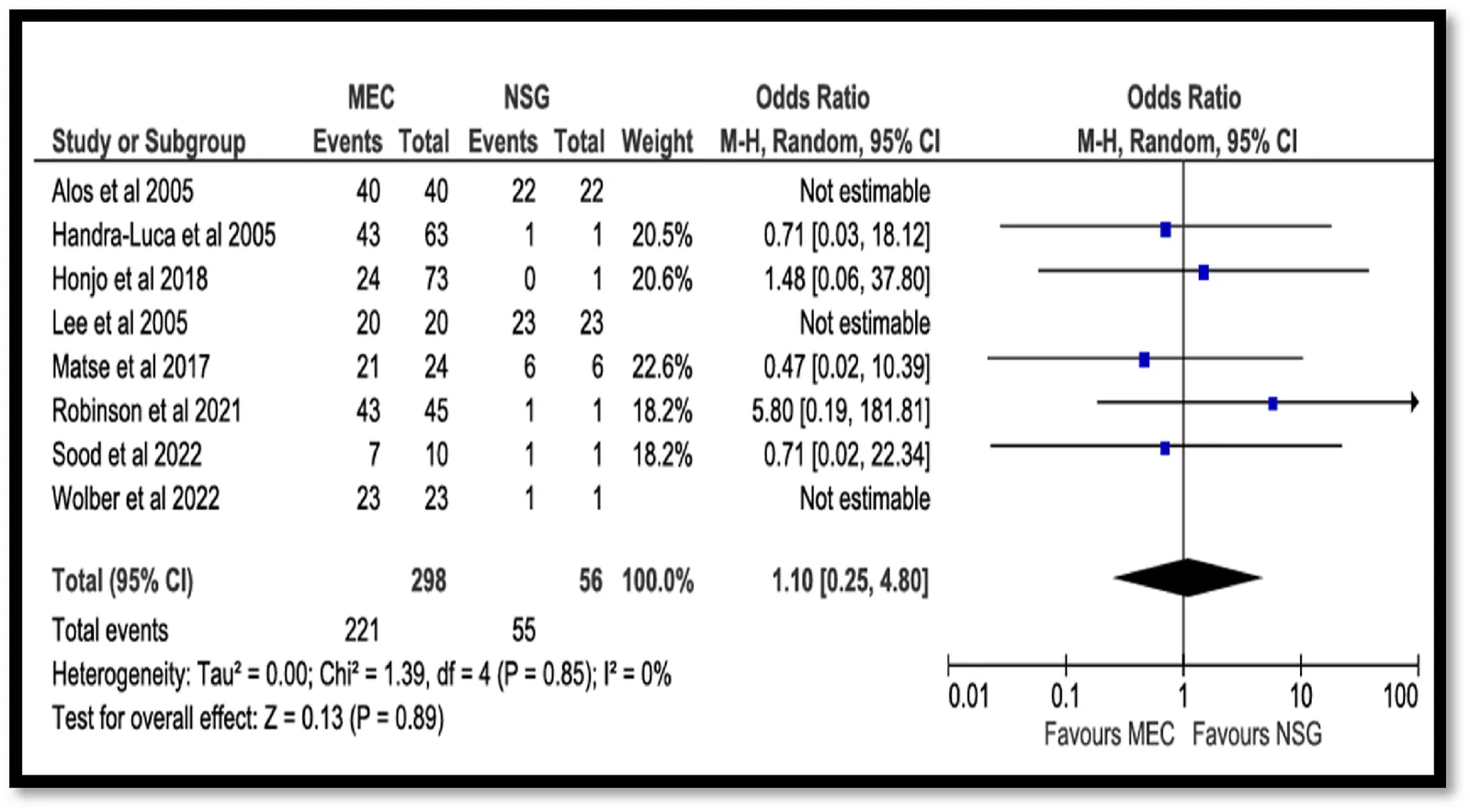
Forest plot showing expression of MUC5AC in MEC.

Eight studies with reduced MUC5AC expression in MEC compared to NSG were chosen for meta-analysis; the random-effects model indicated null heterogeneity (I^2^ = 0%, p = 0.89) and an odds ratio of 1.10 [0.25, 4.80] (p > 0.05) (Fig. 6). In diagnosing MEC, MUC5AC possesses a high sensitivity (74.1%) but relatively low specificity (1.8%) (Table 1).

**Table 1:** Meta-analysis of MUC1, MUC4 and MUC5AC expression in Mucoepidermoid Carcinoma and Pleomorphic Adenoma.

MUCIN	TUMOR	POOLED ODDS RATIO (95% CI)	P VALUE	HETEROGENEITY	SENSITIVITY	SPECIFICITY
MUC1	MEC	2.23, 0.32 – 15.30	0.06	53%	79.2%	18.5%
MUC1	PA	0.35, 0.06, 2.17	0.08	0	82.6%	0
MUC4	MEC	0.85, 0.17, 4.30	0.84	0	81%	0
MUC5AC	MEC	1.10, 0.25, 4.80	0.89	0	74.1%	1.8%

#### 3.5.5 Interpretation of pooled diagnostic performance

Although MUC1, MUC4, and MUC5AC exhibited relatively high pooled sensitivity, their consistently low specificity and non-significant pooled odds ratios indicate limited discriminatory ability as standalone diagnostic biomarkers. Collectively, these findings suggest that mucin expression should be interpreted as an adjunct to conventional histopathological assessment rather than an independent diagnostic test.

## 4 Discussion

The present systematic review evaluated the differential expression of mucins in salivary gland lesions, encompassing 1657 cases across a 30-year span (1993–2023). A diverse group of mucins, including membrane-bound (e.g., MUC1, MUC4) and secretory types (e.g., MUC5AC, MUC5B), along with related glycoprotein epitopes (HMFG1/2, sT, sTn, Tn, T), were analyzed across benign, malignant, non-neoplastic, and normal salivary tissues. The findings offer critical insights into their potential as diagnostic and prognostic biomarkers.^1^^,^^4^^,^^18^^,^^19^^,^^21^^,^^24^, ^25^, ^26^^,^^31^

### 4.1 Mucin expression in benign lesions

PA was the most studied benign lesion, with predominant expression of MUC1 and MUC4. Specifically, MUC1 expression was noted in 79 of 144 cases, localized to both membrane and cytoplasm of excretory ducts, while MUC4 positivity was also recorded in multiple studies.^32^ In accordance with MUC1's established role in epithelial cell polarity and tumour development,^1^^,^^17^^,^^30^ our findings imply a role for MUC1 in epithelial proliferation and ductal differentiation. MUC1 expression was higher in RPA (27/40 cases), which may be associated recurrence risk.^1^^,^^5^ This finding is consistent with that of Soares et al. (2011), who determined that RPA and carcinoma-ex-pleomorphic adenoma (Ca-ex-PA) exhibit higher MUC1 staining than primary PAs.^17^ Warthin tumour (WT) was reported to have a distinctive oncocytic and lymphoid composition, as evidenced by the absence of mucin expression in the aforementioned cases.^30^.

Interestingly, BCA and PCA demonstrated focal expression of MUC1, MUC4, and HMFG, yet again highlighting a possible epithelial lineage connection. Anti-MFG positivity in 8/23 cases of BCAC and 4/11 of BCA further supports a shared histogenetic pathway, although not diagnostically definitive ^20^.

### 4.2 Mucin expression in malignant lesions

MEC, the most represented malignant subtype (n = 833), exhibited strong expression of MUC1 (609 cases) and MUC4 (234 cases), followed by secretory mucins like MUC5AC (167 cases) and MUC5B (56 cases). MUC1's extensive expression correlates with poor prognosis, lymphatic spread, and high-grade histology, as reported by Liu et al. and Siyi et al. ^15^^,^^16^​. Conversely, MUC4 appears associated with lower grade and favorable prognosis, highlighting its inverse prognostic utility compared to MUC1.^9^^,^^21^ This duality supports their potential as prognostic markers: MUC1 as an imminent risk indicator, MUC4 as a protective factor. Moreover, MEC tissues showed increased expression of T, Tn, sT, and sTn antigens, suggesting altered glycosylation profiles in tumor cells.^4^^,^^26^ These antigens' cell-specific expression, reflecting the lineage differentiation of intermediate, mucous, and epidermoid cells, was shown by Fonseca et al. By demonstrating de novo expression of sTn and T in tumour cells, which is absent in normal tissue, Matse et al. further established that these glycol-forms are MEC-specific.^4^^,^^26^

SDC (n = 121) exhibited sporadic expression across MUC2, MUC5AC, MUC5B, and MUC6. While these results were less robust, Simpson et al. highlighted focal MUC positivity in mucin lakes of SDC, underscoring histological variability. The relatively weak expression compared to MEC suggests different mucin processing or lineage.^14^

AdCC displayed MUC1 expression in 26 of 68 cases and minimal MUC2 positivity. These results echo findings from Mannweiler et al. and J-H Lee et al., where AdCC stood out from MEC and ACC employing MUC profiles ^27^^,^^30^​.

ACC demonstrated moderate expression of MUC1 (34/85), MUC4 (44/85), and MUC3 (8/85), while SeC showed overwhelming MUC4 positivity (49/61), confirming MUC4's diagnostic utility.^13^ The specificity and sensitivity of MUC4 for SeC werestriking90.7% and 100%, respectively making it a valuable immunohistochemical marker.

Recent molecular advances have further refined the understanding of salivary gland tumor biology. Mucoepidermoid carcinoma is frequently characterized by CRTC1/3–MAML2 fusion events, whereas secretory carcinoma is defined by the ETV6–NTRK3 fusion.^33^^,^^34^ The observed differential expression of mucins, particularly MUC1 in MEC and MUC4 in secretory carcinoma, may reflect underlying molecular alterations and lineage-specific oncogenic pathways. Although direct mechanistic associations remain incompletely understood, integration of mucin immune-profiles with molecular diagnostics may enhance tumor classification and risk stratification in future studies.

Meanwhile, rare subtypes like MASC, BCAC, EMC, PAC, and IDC showed limited or no significant mucin expression, with IDC, in particular, showing a complete absence across all mucins. These findings may aid in excluding these tumors when mucin positivity is prominent.^10^^,^^13^^,^^20^^,^^33^^,^^35^

### 4.3 Mucin expression in normal and non-neoplastic tissues

Normal salivary gland tissues (n = 147) displayed broad but variable expression across mucins: MUC1 (27), MUC5B & MUC7 (22 each), MUC4 (20), MUC2 (4), and low levels of MUC8 and MUC19. Notably, HMFG1/2 and glycosylated forms (T, Tn, sT, sTn) were also expressed, indicating baseline biosynthesis of these glycoproteins. However, their lower intensity and restricted localization in ductal or acinar cells differentiated them from tumor profiles.^1^^,^^4^^,^^21^^,^^24^ Non-neoplastic lesions exhibited positivity for MUC1 and MUC2, suggesting their presence may not always indicate malignancy and must be interpreted with caution.^18^^,^^19^^,^^36^

### 4.4 Risk of bias and applicability

According to QUADAS-2 analysis, 15 studies were deemed low-risk across all domains, reflecting generally high methodological quality. However, 10 studies had unclear risk and 4 studies had high-risk in-patient selection, potentially due to lack of consecutive sampling or retrospective designs. Index test applicability raised concern in a few studies (e.g., Honjo et al., 2018) due to unclear blinding or interpretive thresholds. Despite this, the majority (≥80%) of studies maintained low risk and good applicability, enhancing confidence in the synthesized evidence. Yet, methodological variability in mucin detection (IHC vs RT-PCR vs ELISA), lack of uniform scoring criteria, and inconsistency in control tissue comparisons introduce heterogeneity.

Development of an internationally standardized scoring system for mucin assessment would improve study comparability, reproducibility, and future meta-analytic synthesis. In the included studies, variations in antibody clones, staining protocols, positivity thresholds, and scoring systems may have influenced pooled effect estimates.

Several included studies were based on relatively small sample sizes, particularly for rare salivary gland tumor subtypes such as secretory carcinoma, epithelial-myoepithelial carcinoma, and basal cell adenocarcinoma. Consequently, findings should be interpreted cautiously, as limited statistical power may affect the reliability and generalizability of observed associations. Several older studies included in this review were conducted using historical salivary gland tumor classifications that predated recognition of entities such as secretory carcinoma. Consequently, classification bias cannot be excluded.

### 4.5 Meta-analysis findings and diagnostic utility

A high level of concordance was found for MUC1 overexpression in MEC and its correlation with poor prognosis, reported by Liu et al. (2014), Siyi et al. (2014), and others. Conversely, discordance arose in the interpretation of MUC1 in PA and RPA, where some studies (e.g., Brieger et al., 2008) found no prognostic value, while others (e.g., Soares et al., 2011) observed significant correlation with recurrence. Such variations may stem from antibody specificity, scoring criteria, or sample size differences. Although multiple studies lacked statistical validation, MUC4 consistently had positive implications in MEC and SeC. The varying results of T, Tn, sT, and sTn markers in a variety of tumors are reflective of their dynamic function and the necessity of standardized methods.

Based on the four forest plots analyzing MUC1 (membranous and cytoplasmic), MUC4, and MUC5AC expression in MEC and PA, the diagnostic utility of these mucins appears limited. MUC1 showed high expression in both MEC and PA, but the pooled odds ratios [MEC: OR = 2.23; PA: OR = 0.35] were statistically non-significant (p > 0.05), with moderate heterogeneity (I^2^ = 53% for MEC) and poor subgroup differences. The moderate-to-high heterogeneity observed across analyses, particularly for cytoplasmic MUC1 expression (I^2^ = 70%), likely reflects substantial methodological differences among studies, including variations in antibody clones, tissue fixation protocols, scoring criteria, positivity thresholds, sample size, and tumor subtype composition. Notably, MUC4, while expressed in a substantial number of MEC cases (sensitivity 81%), exhibited near-zero specificity (0%), and its odds ratio (0.85 [95% CI: 0.17–4.30]) lacked statistical significance. Similarly, MUC5AC demonstrated high MEC sensitivity (74.1%) but extremely low specificity (1.8%), with a pooled OR of 1.10 (p = 0.89).

Although MUC1, MUC4, and MUC5AC demonstrated relatively high sensitivity, their extremely low specificity indicates frequent expression in both tumour and non-neoplastic salivary tissues, thereby limiting their discriminatory ability when used alone. Furthermore, the pooled effect estimates failed to achieve statistical significance, and the wide confidence intervals observed across analyses reflect substantial uncertainty, likely resulting from the limited number of included studies and methodological heterogeneity.

Therefore, although mucins demonstrate diagnostic potential, the available evidence does not justify their use as independent diagnostic markers. Instead, they should be considered supportive biomarkers that complement routine histopathological assessment, particularly in diagnostically challenging cases.

### 4.6 Clinical and diagnostic implications

The observed differential mucin expression underlines their potential use in salivary gland pathology.^37^, ^38^, ^39^, ^40^, ^41^ MUC1 and MUC4 emerged as the most consistently investigated mucins and may serve as useful adjunctive markers when interpreted alongside histopathological and molecular findings rather than in isolation. The distinct profiles across tumor subtypes could assist in challenging diagnoses, such as distinguishing MEC from AdCC or identifying SeC in small biopsies. Moreover, the role of mucin-related antigens (HMFG, sTn) as adjunct biomarkers offers future therapeutic relevance, especially with the advent of glycan-targeted therapies.

### 4.7 Future directions and therapeutic perspectives

Emerging therapeutic strategies targeting mucin-associated signaling pathways may offer novel treatment opportunities. MUC1-directed vaccines, monoclonal antibodies, and antibody-drug conjugates are currently under investigation in several epithelial malignancies. Likewise, inhibition of MUC4-mediated HER2/ERBB signaling pathways has shown promise in preclinical cancer models. Although such approaches have not yet been extensively explored in salivary gland tumors, they represent potential avenues for future translational research and warrant further investigation.^42^^,^^43^

In conclusion, mucins exhibit differential expression and localization across various histological types of salivary gland tumors, providing valuable insights into tumour differentiation, behaviour, and prognosis. Among the evaluated biomarkers, MUC1 and MUC4 demonstrated distinct subtype-specific expression patterns and showed potential as adjunctive markers in salivary gland pathology. Nevertheless, the pooled quantitative evidence remains insufficient to establish their role as reliable standalone diagnostic biomarkers because of limited specificity, non-significant pooled effect estimates, and methodological heterogeneity across the included studies. Future well-designed, large-scale studies employing standardized methodologies are warranted to validate their clinical applicability and further define their diagnostic, prognostic, and therapeutic relevance.

## Consent to participate

Not applicable.

## Data availability

Not applicable (no data).

## Code availability

Not applicable.

## Ethical approval

As no human subjects are included in this review article, no ethical approval or Institutional Review Board was required.

## Author contributions

S.C.- Study conception, Study design, Data acquisition, Quality control of data, Statistical analysis, Manuscript preparation. G.V.- Study design, Data analysis and Interpretation, Statistical analysis, Manuscript editing, Manuscript review. C.P.- Data acquisition, Quality control of data. S.K.- Data acquisition, Manuscript editing. R.A.- Data analysis and Interpretation, Manuscript preparation, Manuscript editing, Manuscript review.

## Human and animal statements

This article does not contain any studies with human participants or animals performed by any of the authors.

## Funding

The authors did not receive support from any organization for the submitted work.

## Declaration of competing interest

The authors declare that they have no known competing financial interests or personal relationships that could have appeared to influence the work reported in this paper.
