# Supplementary material for: Mucin dysregulation in salivary gland tumorigenesis: Diagnostic, prognostic, and therapeutic insights: A systematic review & meta-analysis
Source: J Oral Biol Craniofac Res. 2026 Sep 9;16(5):101533. doi: 10.1016/j.jobcr.2026.101533 (PMC13582008; doi:10.1016/j.jobcr.2026.101533)
Supplement: Supplementary file 1 [file mmc1.docx]

Table 1: Detailed clinical, demographic data, molecular method and molecular diagnostic results of the included articles

| **S. No** | **Country** | **Author & Year** | **Total number of lesions evaluated** | **Type of Mucin** | **Method** | **Absence of expression** | **Localisation of expression** | **Membranous (IHC)** | **Cytoplasmic only (IHC)** | **Cytoplasmic + Membranous (C+M)** | **PCR/**  **ELISA**  **Others** | **Outcome** | **Inference** |
| --- | --- | --- | --- | --- | --- | --- | --- | --- | --- | --- | --- | --- | --- |
| 1 | USA | Williams SB et al 1993 | BCAC (23), BCA (11) | MFG | IHC | BCA (7)  BCAC (15) | luminal areas and their contents of both BCA & BCAC | - | BCA (4)  BCAC (8) | - | - | Anti-MFG reacted with about one third of both BCA and BCAC. | The comparable IHC profiles of BCA & BCAC imply evolvement from similar cell lines and lead us to conclude that distinction between the two is not possible on the basis of these findings. |
| 2 | Portugal | Fonseca I et al 1994 | MEC (32), NSG (12) | Tn, sTn, T, sT | IHC | - | **NSG** =T- myoepithelial cells.  Tn & sTn – mucous glands  **Neoplastic cases –** lumina shows T, sT, Tn & sTn | **-** | **sTn-** mucous cell (20), Intermediate cells (25)  Epidermoid cells (20)  **T-** Intermediate cells (14)  Epidermoid cells (15)  **sT-** mucous cells (4), Intermediate cells (15), Epidermoid cells (20) | **Tn –** mucous cells (7), Intermediate cells (28), Epidermoid cells (28) | - | Mucous cell – only sTn  Epidermoid cell – 57.1%  Intermediate cell – 56.3%  T- 11.5%  sT- 53.6% | The distinct glycosylation pattern suggests although all three cells of MEC are derived from reserve cells of excretory cells yet intermediate cell follow different path of differentiation |
| 3 | Portugal | Simpson R.H.W et al 2003 | SDC (100) | MUC2, MUC 5AC, MUC5B, MUC6, MUC7 | IHC | SDC (96) | MUC2, MUC6, MUC5B – Mucin lakes and tumor cells  MUC5AC & MUC7 - Focal | - | Intracytoplasmic + extracytoplasmic (4) | - | - | Positivity for MUC2, MUC5B, and MUC6 in all cases but only rare staining with MUC5AC and MUC7. Small quantities of mucin have often been described in SDC but not large extracellular mucinous lakes | The poor clinical outcome of the patients in usual-type SDC and emphasizes the importance of differentiating mucin-rich SDC from pure mucinous (colloid) adenocarcinoma |
| 4 | Austria | Mannweiller et al 2003 | PA (21), WT (22), AdCC (26), ACC (13), MEC (9), Non-neoplastic lesion (10) | MUC1, MUC2 | IHC | **PA -**MUC2 (2)  **AdCC –** MUC2 (24)  **ACC-** MUC2 (1) | Luminal borders of inner tubules | - | **PA** – MUC1 (21), MUC2 (19)  **ACC**- MUC1 (13), MUC2 (12)  **AdCC** – MUC1 (26), MUC2 (2)  **WT & MEC**– All cases of MUC1 & 2 | - | - | Strong MUC1 in WT and MC, a moderate in ACC and ACA, and a weak in PA. Strong MUC2 was noted in all WT, moderate expression in MC, and weak expression in PA and ACA. | Mucin immuno histochemistry may prove useful in the classifi cation of salivary gland tumors. The presence of MUC1 may offer new therapeutic strategies in progressive disease |
| 5 | USA | Weed DT et al 2004 | MEC (28) | MUC4 (carbohydrate-dependent -**15H10** & peptide-dependent – **IG8)** | IHC | - | Mucous cell – all positive  Intermediate & squamous cell - variable | - | - | **Low grade** – IG8 (82%), 15H10 (91%)  **Intermediate –** IG8 (67%), 15H10 (100%)  **High –** IG8 (18%), 15H10 (55%) | - | A reduction in MUC4 in high-grade tumors (55% expression) compared with low-grade (91% expression) and intermediate grade (100% expression) tumors. Also correlated with recurrence risk. | MUC4 positivity is associated with reduced risk of death and reduced risk of recurrence and may identify a subset of MEC patients with favorable prognosis. |
| 6 | Japan | Hamada T et al 2004 | PA (40), RPA (9), NSG | MUC1 (Various subtypes),MUC2, MUC4, MUC5AC, MUC6 | IHC | **PA-** MUC2 (38)  MUC4 (35), MUC5AC (36), MUC6 (26), MUC1/HMFG-1 (40) | **NSG-** MUC1 - surface of the ductal cells  MUC2, MUC4, MUC5AC & MUC6- epithelium of intralobular ducts | - | **PA-** MUC1/CORE, (5); MUC1/DF3, (3); MUC1/MY.1E12, (6); MUC1/ glycoprotein, (6);  **RPA -**MUC1/CORE, (1); MUC1/DF3, (4); MUC1/MY.1E12, (2); MUC1/glycoprotein, (2); MUC1/HMFG-1, (1); and MUC6, (1) | | - | MUC1/DF3 (more than 30% of the neoplastic cells stained) in the primary lesions was seen more with recurrence. | Expression of MUC1/DF3 in PA is a useful marker to predict its recurrence. |
| 7 | Spain | Alos L et al 2005 | MEC (40)  NSG (22) | MUC1, MUC2,MUC4,MUC5AC,MUC5B, MUC6, MUC7 | IHC | **NSG –** MUC6 (22) | **NSG –** MUC1 & 4 (ducts, serous & mucous acini), MUC2 & 5AC (ducts), MUC5B (mucous acini), MUC7 (serous acini)  **MEC-**  MUC1 & 4 (all cells), MUC2 & 7 (mucous acini), MUC5A & 5B (mucous & columnar cell), MUC6 (epidermoid & clear cell) | - | **NSG –** MUC1 (13), MUC2 (3), MUC4 (19), MUC5AC (7), MUC5B (22), MUC7 (22)  **MEC-** MUC1 (40), MUC2 (2), MUC4 (38), MUC5AC (29), MUC5B (33), MUC6 (13), MUC7 (2) | - | - | MUC1 was related to high histologic grades, high recurrence, metastasis and a shorter disease-free interval. MUC4 high expression was mainly related to low-grade tumors, lower recurrence rates and a longer disease-free interval | MEC usually express MUC1, MUC4, MUC5AC and MUC5B; less frequently MUC6;and rarely MUC2 and MUC7. MUC1 expression is related to tumor progression and worse prognosis, whereas MUC4 expression is related to a better prognosis. |
| 8 | South Korea | J-H Lee et al 2005 | MEC (20), AdCC (20), ACC (11)  NSG (23) | MUC3, MUC5AC, MUC6 | IHC | **MEC-** MUC3 (20), MUC6 (18)  **AdCC-** All mucins  **ACC-** MUC3 (3), All cases of MUC5AC & 6 | NSG- acinar cells | - | **MEC-** MUC5AC (20), MUC6 (2)  **ACC-** MUC3 (8) |  | - | The combination of MUC3 negative, MUC5AC positive, and MUC6 negative favored MEC. The combination of MUC3 positive, MUC5AC negative, and MUC6 negative favored the ACC. The combination of MUC3 negative, MUC5AC negative and MUC6 negative favored the AdCC. | Positive expression of MUC5AC is very unique to MEC, and that the positive expression of MUC3 is very unique to ACC helping in differential diagnosis |
| 9 | France | Handra-Luca et al 2005 | MEC (63) | MUC1, MUC2, MUC4, MUC5AC | IHC | MUC1 (18), MUC2 (50), MUC4 (13), MUC5AC (20) | **NSG-** MUC1 & 4 (apical membrane of excretory ducts)  MUC2 & 5AC (luminal cells of excretory ducts)  **MEC-** MUC1 & 4 (intermediate, clear, and epidermoid cells), MUC2 & 5AC (intermediate cells) | - | MUC1 (44), MUC2 (13), MUC4 (45), MUC5AC (43) | - | - | Mucin expression in MEC differs from that in salivary glands. Intermediate cells express MUC1 and MUC4 all along their cell surface and MUC2 and MUC5AC in their cytoplasm. | MUC5AC in high-grade tumors can be helpful for distinguishing high-grade MEC from squamous cell carcinoma. While MUC4 is related to tumor differentiation, MUC1 expression indicates a worse prognosis. |
| 10 | Germany | Brieger J et al 2008 | PA (7), RPA (13) NSG | MUC1 | IHC | - | NSG – basal cells | - | PA (7)  No data available about RPA cases | - | - | No statistically significant correlation between MUC1 and recurrence of PA. | No diagnostic or prognostic significance of MUC1 in PA |
| 11 | Mexico | Ponce-Bravo et al 2008 | NSG (15) | HMFG1  HMFG2 | IHC | - | Secretory epithelial cells and ductal cells lining the striated and intercalated ducts in the intraductal secretion material | - | HMFG1& 2 (15)  (Variable intensity) |  | - | Differences in the presence and/or quantity of the studied MUC1 mucin epitopes and demonstrates that both forms of MUC1 | Two main types of myoepithelial cells exist in NSG. HMFG-1 and HMFG-2 proteins may play a role in normal salivary gland function, mainly in the manufacturing and secretion of saliva |
| 12 | Brazil | Soares A et al 2011 | PA (21), RPA (18), Caex-PA (11), TRPA (3)  NSG | MUC1 | IHC | PA (12) | NSG - epithelial cells of the ductal structures. | - | PA (9), RPA (18), TRPA (3)  Expression was stronger in RPA & Caex-PA | - | - | MUC1 in RPA was stronger than PA. MUC-1 expression was significantly higher in carcinoma than in RPA and PA. | MUC-1 is related to the recurrence of PA and malignant transformation of PA |
| 13 | USA | Shemirani N et al 2011 | MEC (19)  NSG (19) | MUC12, MUC13, MUC17, MUC18, MUC19 | RT-PCR | **RTPCR-** MUC12 & 17 (All cases) | - | - | - | - | **RTPCR- NSG-** MUC19 (5)  **MEC**MUC19 (15), MUC13 (3), MUC  **qPCR –** MUC1& 4 four fold increase in Stage 1 vs 4. MUC19 (7). | MUC 19 was found more often in tumor tissue when compared to normal. MUC18 over-expression may be specific in certain carcinomas but does not appear play a significant role in MEC | qPCR measurements of MUC 4 expression can be utilized to predict favorable prognosis and MUC 1 and MUC 19 to predict less favorable prognosis. |
| 14 | China | Siyi L et al 2014 | MEC (62), NSG (10) | MUC1 | IHC | NSG (10) | Data not specified | - | Low to moderate intensity (28), High intensity (34) | - | - | High expression of MUC-1 in high-grade MEC was significantly correlated with lymphatic/regional metastasis and clinic stage & high expression of MUC1 (>75%) is an independent prognostic factor in high-grade MEC | High levels of MUC-1 expression may be a potential marker for worse outcome in high-grade MEC and could be used as a new molecule target to improve outcomes |
| 15 | China | Liu S et al 2014 | MEC (357), NSG (10) | MUC1 | IHC | Normal epithelial tissue, NSG (10), | NSG- cellular membranes and the cytoplasm of epidermoid, intermedi ate, clear, mucous and columnar cells  MEC- Tumor parenchyma tissue. | - | Low intensity (111)  Intermediate (131)  High (115) | - | - | MUC1 in salivary gland MEC is positively correlated with gender, location, grade, stage and lymph node metastasis | Increased expression of MUC1 may be of great value in assessing the development and prognosis of salivary gland MEC, and could be used as a new molecule target to improve outcomes for these patients in the future. |
| 16 | Brazil | Ferreira J et al 2014 | PA (31)  NSG (4) | MUC1 | IHC | PA (9) | Luminal surface of ductal cells | - | PA (22)  NSG (4) | - | - | Strong intensity in 22/31 cases of PA but overall staining was 14% with a median of 9. | Mucosecretory activity does not play a significant role in recurrence of PA |
| 17 | Mexico | Ponce-Bravo S et al 2015 | PA (24) NSG (3) | HMFG1  HMFG2 | IHC | PA (4)- 3 with HMFG1 & 1 with HMFG2 | Ductal, squamous metaplastic and neoplastic myoepithelial cells, keratin pearls and intraductal mucous material. | - | **NSG**- (3)  **PA-** HMFG1- mild intensity (7), moderate (12), strong (3).  HMFG2- mild (5), moderate (11), strong (8) | - | - | Variation in intensity of HMFG1 & 2 observed among PA cases. No significant corelation was stated. | Normal CMyECs are not the ancestor cells during PAs development. MMyECs are responsible in part for the wide histologic variation of these neoplasms. |
| 18 | USA | Lanzel EA et al 2016 | MEC (22), PCA (12), Salivary duct cyst (3) | MUC1, MUC2, MUC4, MUC7 | IHC | **MUC2-** MEC (21), PCA (12), Salivary duct cyst (3)  **MUC4 –** PCA (3)  **MUC7 –** All cases | **MUC1 in MEC –** mucous, intermediate & epidermoid cells |  | **MUC1-**Salivary duct cyst (3)  **MUC2 –** MEC (1) | **MUC1-**MEC (22), PCA (9)  **MUC4 –** MEC (22), PCA (9), Salivary duct cyst (3) | - | Variability in MUC1, MUC4 expression between benign and malignant cases were statistically significant. | Although MUC1, MUC4 was inconclusive, negative expression suggests a benign PCA or salivary duct cyst |
| 19 | UK | Khurram S et al 2016 | ACC (31)  MASC (11) | MUC1, MUC4 | IHC | MUC4- | MUC1- ducts and secretory material MUC4- Luminal and secretory material | - | **ACC-** MUC1 (21), MUC4 (42)  **MASC-** MUC1 (6), MUC4 (6) | - | - | DOG-1, in conjunction with S100 can potentially distinguish between ACC and MASC. MUC1 & 4 are of little help. | MUC1 & MUC4 can serve as an adjunct for diagnosis of MASC. |
| 20 | Germany | Bissinger O et al 2017 | MASC (3) | MUC4 | IHC | MASG (1) | Data not provided | - | MASG (2) | - | - | Moderate to strong intensity of MUC4 is useful for diagnostic workup of MASG | Multimodality based diagnostic workup is needed to distinguish MASG from other salivary gland tumors |
| 21 | Netherlands | Matse JH et al 2017 | MEC (24), NSG (6) | MUC1, MUC4, MUC5AC, MUC5B, Tn, sTn, T | IHC | MUC1 (1), MUC5AC (3) Tn (2), sTn (3), T (3) | **NSG-** T, Tn, sTn- ductal & serous cells  **MEC-** Both mucous and non-mucous cells | - | MUC1 (23), MUC4 (24), MUC5AC (20), MUC5B (23), Tn (22), sTn & T (20) | - | - | MUC1 & 4- loss of topology  MUC5AC, 5B & sTn- de novo expression  Also MUC1, MUC4, Tn, sTn- MEC>NSG | During the development of MEC in the parotid gland, the genes for gel-forming secretory mucins are switched on. Besides these MEC tissues overexpress short oligosaccharides, suggesting that the glycosylation machinery is altered. |
| 22 | Japan | Honjo K et al 2018 | MEC (73), Non neoplastic epithelium | MUC1, MUC2, MUC4, MUC5AC, MUC6 | IHC | MUC1 (19)  MUC2 (66)  MUC4 (39)  MUC5AC (49)  MUC6 (49) | Mainly in cystic component for MUC1 | - | MUC6 (24) | MUC4 (34) | - | Significant relation between mucin pattern with negative MUC4 and positive coexpression of MUC6 | Negative MUC4/6 alone & dually is a reliable marker for shorter DFS/DSS in MEC patients in first 2yrs of treatment. |
| 23 | Japan | Isaka E et al 2021 | MEC (3), NSG (7) | MUC1 and its glycans | IHC + Molecular matrix electrophoresis | NSG (7) | Not specified | - | - | MEC (3) | - | MEC O-glycans – 8 & 14  Normal salivary gland O-glycans -neutral glycans | CA1 in serum can be a confounding factor in assessing MUC1. So exact glycan structure of MUC1 needs to be studied while diagnosing MEC. |
| 24 | Germany | Schicht M et al 2021 | NSG (6) | MUC8 | ELISA, Immunoblot |  | Intralobular excretory ducts. Submandibular- luminal cell pole of the epithelial cells.  Parotid- around individual nuclei | - | - | - | MUC8 (6) | The mean MUC8 conc was 71.06 ng/mL in female and 33.21ng/mL in males. MUC8- 15times higher with salivary stone pathology. | MUC8 concentration is gender dependent and is highly secreted in patients with salivary stone |
| 25 | South Africa | Robinson L et al 2021 | MEC (45) | MUC1, MUC2, MUC4 MUC5AC | IHC | MUC2 (44), MUC4 (2), MUC5AC (2) |  | - | MUC1 (45), MUC2 (1), MUC4 (43), MUC5AC (43) but with variable intensity | - | - | MUC1 overexpression - reliable marker of high-grade MECs, MUC4 overexpression - more indicative of low-grade tumours  MUC2- excluding MEC as a diagnosis. No statistically significant association between MUC expression & MAML2 rearrangement status. | MECs show an altered MUC expression pattern that can be used for diagnostic purposes and to aid in establishing a more accurate tumour grade. |
| 26 | Germany | Wolber P et al 2022 | MEC (23), AdCC (22), SDC (21), ACC (10), ADNOS (9), EMC (7), SeC (7), Others (8)  TOTAL=107 | MUC1, MUC5AC, MUC16 | IHC | MUC1 – (26)  MUC16- (27)  MUC5AC- (97) | MUC1 & MUC16 – Apical, membranous & cytoplasmic staining  MUC5AC- |  | MUC5AC Mean score- 5.3±23.3 | MUC1 –mean score 109.1±107.4  MUC16- mean score 74.6±104.4 | - | **MUC1 Score**- SDC>MEC>SeC>ADNOS>EMC>ACC>AdCC  **MUC16 Score-**  MEC>ADNOS>ACC>SDC>AdCC>EMC>SeC  **MUC5AC Score-**  MEC>SDC>ADNOS | MUC1 – highly correlated with LVI, PNI, ENE thus qualifying as therapeutic target in metastatic SDC.  MUC16 – Most useful in high grade MEC |
| 27 | India | Sood N et al 2022 | MEC (10) | MUC1, MUC2, MUC5AC | IHC | MUC2 (3), MUC5AC (3) - Intermediate & epidermoid cells of all cases | MUC1-Apical membrane of excretory ducts  MUC2 & MUC5AC - luminal cells lining excretory ducts | Goblet cell component of all cases | - | MUC1 (10)  MUC2 (7)  MUC5AC (7) | - | MUC1 – All membranous to focal cytoplasmic localization  MUC2 - cytoplasmic localisation in 40% of mucinous cells and was minimal in other cells  MUC5AC- strong apical membranous in goblet cells | MUC1 - reliable marker for all cell types of MEC (unrelated to grade)  MUC5AC – MEC with lymphoid infiltrate |
| 28 | Japan | Sugiura T et al 2023 | MEC (3), NSG (7) | MUC1, sTn | IHC | NSG - sTn (100%),  MUC1 serous & mucous acini (100%) | NSG - apical membranes | - | **MEC** – MUC 1 All 3 cases in all cell types  **NSG** – MUC1 1 case each in mucous cell & non-mucous cell | - | - | MUC1 was diffusely detected at MEC but was detected at apical membranes in NSGs. C2GnT-1 was expressed at the MUC1‑positive in MEC but not in NSG. MEC cells produced MUC1 modified with α2,3‑linked sialic acid containing core‑2 O-glycans | MUC1 modified with α2,3-linked sialic acid-containing core-2 O-glycans in MEC was distinctively expressed in mucous cells and non-mucous cells. |
| 29 | Germany | Taverna C et al 2020 | SeC (54), ACC (20), PAC (21), IDC (30) | MUC4 | IHC | SeC (5)  IDC (30)  ACC (20)  PAC (21) | Data not available | - | SeC (49) | - | - | MUC4 as a sensitive (90.7%) and specific (100%) marker for SeC, with high positive (100%) and negative (93.4%) predictive values. | MUC4 may be used as a surrogate for SeC in limited biopsy material and in cases with equivocal morphology. |

**BCAC**- basal cell adenocarcinoma, **BCA**- basal cell adenoma, **MFG-** milk fat globulin, **IHC**- immunohistochemistry, **MEC**- mucoepidermoid carcinoma, **NSG**- normal salivary gland, **T**- simple mucin-type carbohydrate antigen T, **Tn-** simple mucin-type carbohydrate antigen Tn, **sT-** simple mucin-type carbohydrate antigen sialosyl-T, **sTn-** simple mucin-type carbohydrate antigen sialosyl-Tn, **SDC-** salivary duct carcinoma, **MUC-** mucin, **PA-** pleomorphic adenoma, **WT-** warthin tumor, **AdCC-** adenoid cystic carcinoma, **ACC-**acinic cell carcinoma, **RPA-** recurrent pleomorphic adenoma, **HMFG-** Human milk fat globule membrane, **Caex-PA-** carcinoma ex-pleomorphic adenoma, **TRPA-** pleomorphic adenoma with malignant transformation, **RT-PCR-** reverse transcriptase-polymerase chain reaction, **qPCR-** quantitative polymerase chain reaction, **MMyEC-** modified myoepithelial cell, **CMyEC-** classic myoepithelial cell, **PCA-** papillary cystadenoma, **MASC-** mammary analogue secretory carcinoma, **ELISA-** enzyme linked immunosorbent assay, **ANOS-** adenocarcinoma not otherwise specified, **EMC-** epithelial-myoepithelial carcinoma, **SeC-** secretory carcinoma, **PAC-** polymorphous adenocarcinoma, **IDC-** intraductal carcinoma
